# Supplementary material for: Effects of Eggshell Membrane on Keratinocyte Differentiation and Skin Aging In Vitro and In Vivo
Source: Nutrients. 2021 Jun 22;13(7):2144. doi: 10.3390/nu13072144 (PMC8308305; doi:10.3390/nu13072144)
Supplement: Supplementary file 1 [file nutrients-13-02144-s001.zip › nutrients-1240674-supplementary.pdf]

Table S1. Amino acids composition of three ESMs

Amino acids	pESM	hESM	eESM
Aspartate	0.027 ± 0.004	0.849 ± 0.038	0.176 ± 0.003
Glutamate	0.027 ± 0.002	1.272 ± 0.057	0.588 ± 0.011
Asparagine	0.003 ± 0.001	0.016 ± 0.001	0.088 ± 0.001
Serine	0.009 ± 0.001	0.748 ± 0.033	0.107 ± 0.002
Glutamine	ND	0.042 ± 0.002	0.017 ± 0.000
Histidine	ND	ND	0.605 ± 0.005
Glycine	0.043 ± 0.034	3.614 ± 0.158	1.177 ± 0.022
Threonine	0.013 ± 0.002	0.007 ± 0.001	0.207 ± 0.005
Citrulline	ND	0.075 ± 0.003	0.282 ± 0.004
Arginine	0.009 ± 0.001	0.011 ± 0.000	0.519 ± 0.008
b-Alanine	0.003 ± 0.000	ND	ND
Alanine	0.010 ± 0.002	2.203 ± 0.092	1.012 ± 0.017
Taurine	ND	0.003 ± 0.000	0.010 ± 0.000
Tyrosine	0.005 ± 0.000	0.155 ± 0.007	0.480 ± 0.007
Valine	0.010 ± 0.001	0.214 ± 0.010	2.006 ± 0.035
Methionine	0.003 ± 0.000	0.210 ± 0.008	0.871 ± 0.012
Tryptophan	0.017 ± 0.001	0.276 ± 0.012	0.294 ± 0.005
Phenylalanine	0.017 ± 0.001	0.144 ± 0.007	0.471 ± 0.007
Isoleucine	0.005 ± 0.000	0.029 ± 0.013	0.684 ± 0.011
Leucine	0.010 ± 0.001	0.290 ± 0.013	1.390 ± 0.023
Ornithine	ND	0.778 ± 0.035	0.634 ± 0.049
Lysine	ND	0.257 ± 0.011	0.312 ± 0.006
Proline	ND	1.185 ± 0.046	0.141 ± 0.006
Total Amino acids	0.210	12.421	12.178

100 mg of three types of ESMs were dissolved in 1 ml of water and 300 µl of aliquot was mixed with 300 µl of 1.5 M $HClO_4$ and 150 µl of 2 M K_2CO_3. After centrifugation at 13,500 rpm for 10 min, 300 µl of the supernatant was mixed with the same volume of hexane and vortex well. After centrifugation with the above condition, the water layer was utilized for amino acid analysis with ultra-high performance liquid chromatography (Nexera X2 series, Shimadzu, Kyoto, Japan). Data are means ± SE, n = 3 and expressed as mg/100mg. ND means that the amino acid was not detectable in our method.

Table S2. Primer sequences

Gene		Primer sequence (5′-3′)
For human		
CTSV	*Sense*	ACCGCGGACGTCTGTAATCT
	Antisense	TTCAAAACCAGCAGCCGTCG
DSG1	*Sense*	ACAGAGAGCAATACGGCCAG
	Antisense	ATGTTGCACTCACATTCCGC
FLG	*Sense*	TCGGCAAATCCTGAAGAATCCA
	Antisense	TGTGGTCTATATCCAAGTGATCCA
IVL	*Sense*	GAGTCTGGTTGACAGTAGCTT
	Antisense	GGAGGAACAGTCTTGAGGAGC
KLK5	*Sense*	CGAGGATGCTTACCCGAGAC
	Antisense	CCTGGCAGGAGTCTCTACCT
KLK7	*Sense*	CCCCTGCAGATCCTACTGCTA
	Antisense	ACATGGGGCGCCATCAATAAT
KRT1	*Sense*	TGGACCTTCAGGCCAAACTT
	Antisense	TGCATCTGAGACAACTCTGCTT
KRT5	*Sense*	GAGATCGCCACTTACCGCAA
	Antisense	TGCTTGTGACAACAGAGATGT
KRT10	*Sense*	TCCCAACTGGCCTTGAAACA
	Antisense	TGAGAGCTGCACACAGTAGC
KRT14	*Sense*	CAGTCATCCAGAGATGTGACCT
	Antisense	TTGCCATCGTGCACATCCAT
SPINK5	*Sense*	AATGCAAAGGATGAGTGCAGTG
	Antisense	TCTCTCTAGGGCAGATGAGTTC
TRPV3	*Sense*	AACCTCACCAGCCATGAAAGC
	Antisense	TGTGCACTCTTCTTTGTGGGG
TRPV4	*Sense*	GATCTTTCAGCACATCATCC
	Antisense	GGTCATAAAGCGAGGAATAC
TRPV6	*Sense*	GCTCTATGAGGGTCAGACTGC
	Antisense	ATGAGGTTGCAGGGACTACG
For mice		
Col1a1	*Sense*	ACCTACAGCACCCTTGTGGA
	Antisense	GGTGGAGGGAGTTTACACGA
Ctgf	*Sense*	CAAACAAATGCTGTGCAGGT
	Antisense	AGCAAGCACTTCCTGGTAGG

Tert	*Sense*	CAGCCATACATGGGCCAGTTC
	Antisense	ACAGGCTGCTGCTGCTCTCA
Tgfb1	*Sense*	GGCACCGGAGAGCCCTGGATA
	Antisense	AATGTACAGCTGCCGCACACAGC
Tgfb2	*Sense*	CCTTTTCTGCGTCAGTGTGA
	Antisense	GCTTTTGAACGGCAAAGAGA
Tgfb3	*Sense*	GCACTGCCTGGAATTAAGGA
	Antisense	AAGAAGGAAGGCAGGAGGAG
Trpv3	*Sense*	CACGAGAGGCTTCCAGTCTATG
	Antisense	CGCTACTCCAAATCCAAGTAAGAA
Trpv4	*Sense*	GTCCAAGGAGAGCAAGCACA
	Antisense	TCAGGAACACAGGGAAGGAAC
Trpv6	*Sense*	CATCATCGCCACACTGCTC
	Antisense	GGTAGCTTCCGCTCTAACATCAC
Pdgfb	*Sense*	TGGATCATCATGAGCGATGT
	Antisense	CCATGGCACTAACAAAGCAA
Gapdh	*Sense*	GGTGAAGGTCGGTGTGAACG
	Antisense	CTCGCTCCTGGAAGATGGTG

Table S3. Major gene list of "Growth of epithelial tissue"

Gene symbol	Gene name	Fold change (KOE vs KO)
Adipoq	Adiponectin	4.36
Adora2b	Adenosine A2b Receptor	2.10
Akt1	AKT Serine/Threonine Kinase 1	2.12
Braf	B-raf proto-oncogene, serine/threonine kinase	2.02
Ccl5	C-C motif chemokine ligand 5	3.59
Col1a1	Collagen type I alpha 1 chain	3.31
Col1a2	Collagen type I alpha 2 chain	2.05
Col18a1	Collagen type XVIII alpha 1 chain	1.76
Cul4A	Cullin 4A	1.82
Edf1	Endothelial differentiation related factor 1	2.08
Egfl7	EGF like domain multiple 7	1.87
Eya1	EYA transcriptional coactivator and phosphatase 1	3.12
Fabp4	Fatty acid binding protein 4	2.25
Fgf1	Fibroblast growth factor 1	2.29
Gpc1	Glypican 1	1.78
Has2	Hyaluronan synthase 2	1.98
Itga4	Integrin subunit alpha 4	2.24
Kcnk2	Potassium two pore domain channel subfamily k member 2	1.94
Kitlg	KIT ligand	1.81
Map2k5	Mitogen-activated protein kinase kinase 5	2.03
Map2k7	Mitogen-activated protein kinase kinase 7	2.19
Nfatc1	Nuclear factor of activated T cells 1	1.79
Parp1	Poly(ADP-ribose) polymerase 1	1.95
Pde5a	Phosphodiesterase 5A	2.14
Pgf	Placental growth factor	1.85
Pgr	Progesterone receptor	1.77
Plxnb3	Plexin B3	2.47
Postn	Periostin	2.17
Runx1	RUNX family transcription factor 1	1.95
Snai1	Snail family transcriptional repressor 1	1.94
Sox4	SRY-box transcription factor 4	1.91

Sptbn1	Spectrin beta, non-erythrocytic 1	1.85
Wnt5a	Wnt family member 5a	1.88
Wnt7a	Wnt family member 7a	1.54

In this category, 50 genes were listed up as the expression variation gene. Of these, we showed 34 genes which were expected to upregulate the category.
